# How Can We Support the Use of Oral PrEP Among Young Women who Sell Sex? A PrEP Cascade Analysis

**DOI:** 10.1097/QAI.0000000000002733

**Published:** 2021-05-19

**Authors:** B. Hensen, F. Machingura, J. Busza, I. Birdthistle, S.T. Chabata, T. Chiyaka, S. Floyd, G. Jamali, P. Mushati, J. Hargreaves, F.M. Cowan

**Affiliations:** a Faculty of Infectious and Tropical Diseases, London School of Hygiene and Tropical Medicine, London, United Kingdom;; b Centre for Sexual Health and HIV/AIDS Research (CeSHHAR) Zimbabwe, Harare, Zimbabwe;; c Centre for Evaluation, London School of Hygiene and Tropical Medicine, London, United Kingdom;; d Faculty of Epidemiology and Population Health, London School of Hygiene and Tropical Medicine, London, United Kingdom; and; e Faculty of Clinical Sciences and International Public Health, Liverpool School of Tropical Medicine, Liverpool, United Kingdom.

**Keywords:** HIV infections, HIV prevention, oral pre-exposure prophylaxis, women, female sex workers, Zimbabwe

## Abstract

We constructed self-reported pre-exposure prophylaxis (PrEP) cascades and explored factors associated with and barriers to PrEP use to inform efforts to support PrEP use among young women who sell sex.

**Methods:**

Using self-reported data from HIV-negative young women who sell sex enrolled into a cohort study using respondent-driven sampling in Zimbabwe, we constructed PrEP cascades assessing knowledge of, ever offered, ever used, and current PrEP use in 2017 and 2019. We used logistic regression to examine factors associated with PrEP use by 2019. Through qualitative interviews with 43 women enrolled in the cohort, we investigated barriers to PrEP use.

**Results:**

At enrollment, 50% of women had heard of PrEP, 12% had ever been offered PrEP, and 7% ever used PrEP. Over time, all cascade domains: 96% of women had heard of and 55% reported an active offer of PrEP. Among women retained in the study in 2019 (56%; n = 538), 34% ever took PrEP by 2019. PrEP use was associated with, at enrollment, reporting more clients in the past month (10+: 45% vs 1–3: 27% adjOR = 1.71 95% CI: 1.06 to 2.76), duration of selling sex (24% <2 years vs 38% 2–3 years; adjOR = 0.51 95% CI: 0.32 to 0.83), and having visited a female sex worker program in the past 12 months (55% vs 27%; adjOR = 2.92 95% CI: 1.91 to 4.46). Qualitative interviews revealed fear of disclosing sex work, HIV-related/ART-related stigma, and (opportunity) costs of accessing PrEP as barriers to use.

**Conclusion:**

PrEP use was associated with factors known to increase HIV risk. Fear of stigma, disclosure, and supply-side barriers need to be addressed to increase women's ability to use PrEP.

## Abstract 2

Supplemental Digital Content is Available in the Text.

## INTRODUCTION

Adolescent girls and young women aged 15–24 years account for <26% of new HIV infections in eastern and southern Africa.^1^ Among this population, young women who sell sex (YWSS) are at particularly high risk of HIV, reporting higher numbers of partners and less condom use than their peers not involved in sex work and having poorer access to health services than older female sex workers (FSWs).^2–4^ Reducing HIV incidence among YWSS requires combination prevention that is responsive to their needs and addresses barriers to their service use.^5^

Oral pre-exposure prophylaxis (PrEP) is efficacious when taken as prescribed.^6,7^ As YWSS often struggle to negotiate condom use, PrEP offers women more choice and control over their sexual health.^8^ For PrEP to be effective at population levels, individuals must have high demand; PrEP services need to be available, accessible, and acceptable; and those who initiate PrEP must adhere during periods of use. To maximize the potential impact of PrEP, cascades and continuums have been used to identify gaps in delivery and use,^9^ target programs to support initiation, and optimize adherence and continued use.^10^

In Zimbabwe, YWSS were among the target population for the DREAMS initiative, which sought to reduce HIV incidence by 40% among adolescent girls and young women in 10 African countries.^11,12^ In districts where DREAMS funding was available, PrEP availability was a key service for YWSS combined with efforts to increase demand and support use. We previously showed that although it is plausible that DREAMS may have contributed to reduced HIV risk among YWSS, it did not achieve 40% reduction in HIV incidence.^13^ We also showed that new HIV infections did not differ by ever-reporting PrEP use in 2019. To inform efforts to support PrEP use among YWSS, we constructed a PrEP cascade to investigate and understand reasons for gaps in PrEP use and explored whether factors likely to influence demand for and opportunities to access PrEP were associated with uptake.

## METHODS

### Study Location and Population

Data were collected from YWSS aged 18–24 years residing in two Zimbabwean cities selected for the DREAMS initiative. In each city, the national FSW HIV prevention program, “*Sisters with a Voice*” (Sisters), offered HIV testing, condom promotion and distribution, community mobilization, and referral to legal advice through static clinics. At Sisters, YWSS were offered referrals to other DREAMS partners offering additional HIV prevention services, including oral PrEP and social protection.^14^

### Data Collection

Respondent-driven sampling (RDS) was used to recruit YWSS to the DREAMS impact evaluation.^15^ Described elsewhere, the evaluation estimated the impact of DREAMS on HIV incidence among women recruited through RDS and followed up 24-month post-enrollment. Before RDS, YWSS identified through mapping and representative of the typology of sex work in each city were invited to act as “seeds”.^15,16^ To initiate RDS, these “seed” participants were given two vouchers to recruit YWSS, whom they knew and knew sold sex. Eligible participants were given two vouchers to recruit two further women. This process continued over six recruitment waves.^15^ YWSS aged 18–24 years who sold sex were eligible, defined as sex in exchange for material goods/money where the sex would not happen in the absence of the exchange.

### Behavioral Survey

Consenting women completed an interviewer-administered questionnaire and were offered HIV-testing services.^15^ All women received the result of their HIV test. Women were followed up 12- and 24-months postenrollment (2018 and 2019, respectively), asked to complete the same questionnaire, and offered HIV testing as at enrollment. The questionnaire elicited women's demographics, history of selling sex, sexual behaviors, access to HIV prevention services, and engagement with the broader package of services available through DREAMS.

### Qualitative Interviews

Between September 2017 and November 2019, 43 DREAMS participants from among the women recruited through RDS were purposively selected for interview. Women were selected for diversity in age and levels of participation across the range of DREAMS services. Interviews were conducted 12 and 24-months post-enrollment and explored motivations for engaging with DREAMS services and any challenges encountered during participation in DREAMS. A shorter version of the interview topic guide was piloted before RDS. We interviewed 19 YWSS selected as “seeds” for the RDS and explored feasibility of asking about their sexual relationships, involvement in selling sex, engagement with care, barriers to accessing HIV prevention and treatment, and interest in activities to be offered under DREAMS. Interview guides were subsequently expanded to elicit perceptions and experiences and explore if and how involvement in DREAMS affected their health, behavior, and well-being. Sixteen respondents were interviewed >1 time to understand changing experiences and perceptions. Two female social scientists conducted interviews, which lasted 30–60 minutes and were audio recorded, in Shona or Ndebele.

### Outcomes and Explanatory Variables

The cascade used in this analysis was informed by the framework proposed by Schaefer et al,^10^ which conceptualizes that demand for, access to, and use of/adherence to an HIV prevention product are influenced by individual-level and structural-level factors. Self-reported outcomes of interest for the PrEP cascade included ever having heard of, ever being offered, ever using PrEP, and current PrEP use. For women followed up in 2018 and/or 2019, women were defined as having heard of, ever being offered, or ever using PrEP by 2019 if they reported these outcomes at any point during the study. A further outcome of interest was HIV seroconversion.

To understand gaps across the PrEP cascade, we described domains (measured in 2019) believed to influence each step. For having heard of PrEP, measures included knowledge of other prevention measures (sex with one HIV-negative partner, consistent condom use, and medical male circumcision). For this step, we also explored perceptions of other YWSS′ willingness to take PrEP and whether PrEP was believed to make it easy for YWSS to protect themselves from HIV. Measures for potential reasons for gaps in ever being offered PrEP included: attending a Sisters clinic and testing for HIV in the past 6 months. Measures explored as underlying gaps in ever and current use of PrEP included: perceived support for PrEP use from friends/peers, family and sexual partners, condomless sex with regular partners and clients in the past month, and self-perceived risk of HIV in the next 12 months.

Explanatory variables explored for their association with PrEP use included those likely to influence decisions and opportunities to initiate PrEP.^17,18^ These variables, measured at enrollment, included age, marital status, educational attainment, being at risk of common mental health disorders, measured using the Shona Symptom Questionnaire,^19^ whether women self-identified as a FSW, age at which women started and duration of selling sex, client numbers in the past month, self-perceived HIV risk in the next 12 months, knowledge of other HIV prevention measures, perceived support for PrEP use, and attendance at a Sisters clinic in the 12 months before enrollment. In addition, we explored whether ever being offered PrEP by 2019 and women's identification as an FSW between 2017 and 2019 were associated with PrEP use.

### Data Analysis

We restricted analyses to women testing HIV-negative at enrollment. First, we described enrollment characteristics and behaviors of all women and of those retained in the 2019 follow-up survey. Among these, we described the number and proportion of new HIV infections by PrEP use and explored whether steps in the cascade were associated with seroconversion.

Second, using data from all the three surveys, we constructed PrEP cascades for 2017 and 2019. Using the framework proposed by Schaefer et al,^10^ we estimated levels of having heard of, ever offered, ever using, and current use of PrEP at enrollment (2017) and by 2019. For each step, we described potential reasons for gaps, as described, and, adjusting for age and site, explored whether these factors differed within each step. To further understand gaps in the cascade, we conducted thematic content analysis of qualitative interviews with the specific aim of interrogating each stage of the prevention cascade as perceived and experienced by women participating in DREAMS. This involved recoding transcripts into broad codes relevant to each step of the cascade. Data were transcribed and translated into English and imported into NVivo-12. Thematic analysis was achieved by rereading transcripts to identify emerging themes and reconciliation of codes after iterative discussions. We present data on women's understanding of PrEP, where they accessed services, and narratives around choosing to initiate PrEP and subsequent (dis)continuation.

In our third step, we used logistic regression to explore the association between the explanatory variables described and PrEP use by 2019. Our crude models adjusted for age at enrollment and included a fixed effect for site. All variables were explored in adjusted analyses, in which we adjusted for variables associated with PrEP use at *P* < 0.10 level in crude analyses. Adjustment followed the distal–proximal framework used in an analysis of enrollment data,^2^ and as such, we did not adjust for variables considered proximal to the outcome relative to the variable of interest. As our analysis used data at follow-up and half the women were not retained, we did not RDS weight the data. This aligns with our analysis of the primary outcome.^13^

### Ethics

The Medical Research Council Zimbabwe (MRCZ/A/2085) and the London School of Hygiene & Tropical Medicine (11835) approved the study. Written informed consent was obtained from all women before participation.

## RESULTS

### Enrollment Characteristics and Retention

Among 963 HIV-negative women enrolled in the study, 20.5% (n = 197) were aged 18 years and >50% considered themselves FSWs (66.5%, n = 633) and reported ≥3 clients in the past month (57.2%, n = 551). Most considered their risk of HIV in the next 12 months to be low/small (62.8%, n = 501). Half had knowledge of other HIV prevention measures (48.3%; n = 465). More than half the women were retained in the study in 2018 (54.4%; n = 524) and 2019 (55.9%; n = 538). Retention in 2019 was higher among women who, at enrollment, reported selling sex for ≥4 years and started selling sex at the age of 10–14 years (Table 1).

**TABLE 1.** Characteristics and Behaviors of Women Testing HIV-Negative at Enrollment (N = 963) and Retention of These Women in 2019 (N = 538)

	2017	2019			*P* *
	Distribution of Characteristics and Behaviors (N, Column %)	Distribution of Enrollment Characteristics and Behaviors (N, Column %)		Retention by Enrollment Characteristics and Behaviors (Row %)	
Overall	963	538	100%	55.9%	
Age at enrollment (yr)					
18	197 (20.5)	109	20.3%	55.3%	0.69
19	164 (17.0)	99	18.4%	60.4%	
20	112 (11.6)	56	10.4%	50.0%	
21	133 (13.8)	75	13.9%	56.4%	
22	149 (15.5)	80	14.9%	53.7%	
23	155 (16.1)	91	16.9%	58.7%	
24	53 (5.5)	28	5.2%	52.8%	
Marital status at enrollment					
Single/never married	668 (69.4)	361	67.1%	54.0%	0.31
Married/cohabiting	21 (2.2)	12	2.2%	57.1%	
Divorced	270 (28.0)	164	30.5%	60.7%	
Widowed	4 (0.4)	1	0.2%	25.0%	
Highest level of education attained					
No education/incomplete primary	28 (2.9)	15	2.8%	53.6%	0.70
Complete primary education	61 (6.3)	32	6.0%	52.5%	
Form 1–3	393 (40.8)	216	40.2%	55.0%	
Form 4–6	471 (48.9)	271	50.4%	57.5%	
College, cert, or degree	10 (1.0)	4	0.7%	40.0%	
Whether food insecure past mo					
No	418 (43.4)	229	42.6%	54.8%	0.71
Yes	545 (56.6)	309	57.4%	56.7%	
Experienced common mental health illness					
No	595 (61.8)	331	61.5%	55.6%	0.86
Yes	368 (38.2)	207	38.5%	56.3%	
Self-identifies as an FSW					
No	319 (33.5)	167	31.4%	52.4%	0.15
Yes	633 (66.4)	365	68.6%	57.7%	
Age started selling sex					
10–14	36 (3.7)	26	4.8%	72.2%	0.02
15–17	424 (44.1)	250	46.6%	59.0%	
18–19	297 (30.9)	150	27.9%	50.5%	
20–24	205 (21.3)	111	20.7%	54.2%	
Duration of selling sex					
<2	270 (28.1)	136	25.3%	50.4%	0.05
2–3	420 (43.7)	236	43.9%	56.2%	
4+	272 (28.3)	165	30.7%	60.7%	
No of partners sold sex to in the past mo					
1–3	412 (42.8)	219	40.7%	53.2%	0.31
4–9	295 (30.6)	173	32.2%	58.6%	
10+	256 (26.6)	146	27.1%	57.0%	
Perceived HIV risk†					
No chance: no possibility of becoming infected	194 (22.8)	94	19.8%	48.5%	0.04
Small chance: could happen but not likely	307 (36.1)	178	37.5%	58.0%	
Moderate chance: some possibility of becoming infected	121 (14.2)	71	15.0%	58.7%	
High chance: likely to become infected	175 (20.6)	96	20.2%	54.9%	
I do not know	53 (6.2)	36	7.6%	67.9%	
Knowledge of prevention measures‡					
No	498 (51.7)	278	51.7%	55.8%	0.97
Yes	465 (48.3)	260	48.3%	55.9%	

* Wald test, adjusted for site.

† 113 missing data or do not know response.

‡ Knowledge that having sex with one HIV-negative partner, consistent condom use, and male circumcision are measures that can reduce the risk of HIV infection for individuals.

By 2019, 5.8% (n = 31) of women seroconverted, among whom 38.7% (n = 12) reported ever using PrEP between 2017 and 2019. Adjusting for age and site, there was little evidence for an association between steps in the cascade and HIV seroconversion (Suppl Table 1, http://links.lww.com/QAI/B675).

### Cascade Step 1: Ever Heard of PrEP

**FIGURE 1.**
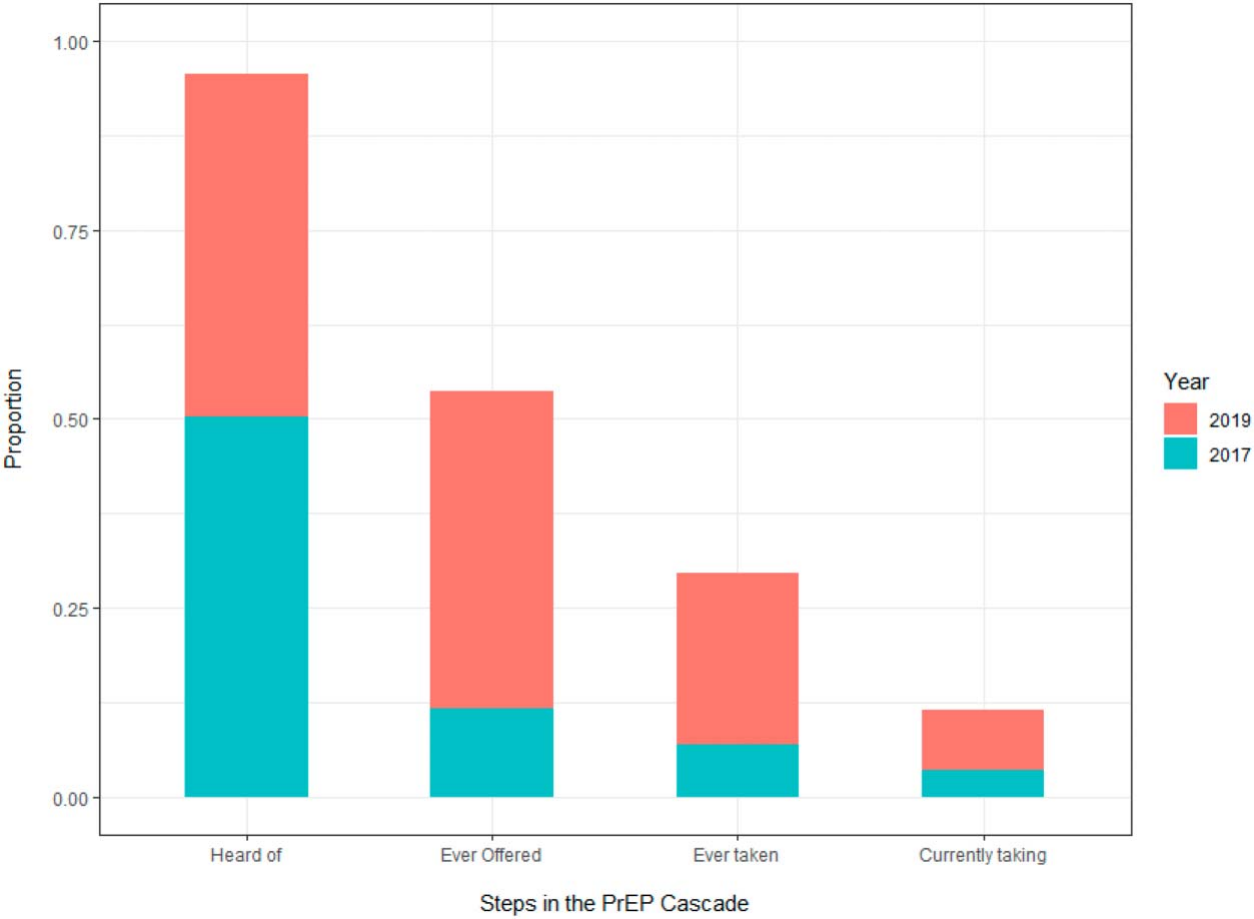
Self-reported PrEP use cascades among young women who sell sex in Zimbabwe, 2017–2019.

Over time, all domains of the cascade increased (Fig. 1). By 2019, most women had heard of PrEP (95.7%; n = 514) compared with half (50.3%; n = 481) in 2017. Never having heard of PrEP was higher among women who disagreed (22.7%; n = 5/22) that PrEP would make it easier for women to protect themselves from HIV than among women agreeing with the statement (5.1% n =16/316; Table 2). In qualitative interviews (Table 3), women described how they heard of and informed their friends about PrEP:

I know about it, when you go to Sisters [Clinic] they always talk about it [PrEP] and it feels like a song (chuckles). I was supposed to go collect it [PrEP] on Wednesday but I did not go…. I did not have bus fare (22 years) BYOLN03R3.

**Table 2.** Potential Reasons Underlying Gaps in the Cascade Among Women Retained in, and as Reported in, the 2019 Survey (N = 538)

	Distribution of Variables (N, Column %)	Number of Women by Gaps in the Cascade (n, Row %)	
Potential Reasons Underlying Gaps in Demand for PrEP by 2019			
		Had Not Heard of PrEP (n = 23)	*P* *
Knowledge of other HIV prevention measures in 2019			
No	211 (39.3)	12 (5.7)	0.18
Yes	326 (60.7)	11 (3.4)	
Perceived risk of HIV in the next 12 months (N = 483)			
None/small	397 (82.0)	17 (4.3)	0.39
Medium/high risk	87 (18.0)	6 (6.9)	
Most young women like me would be willing to take PrEP			
Strongly agree	123 (23.0)	2 (1.6)	0.34
Agree	283 (53.0)	14 (5.0)	
Disagree	100 (18.7)	7 (7.0)	
Strongly disagree	28 (5.3)	0	
PrEP would make it easier for women like me to protect ourselves			
Strongly agree	193 (36.1)	2 (1.0)	0.004
Agree	316 (59.2)	16 (5.1)	
Disagree	22 (4.1)	5 (22.7)	
Strongly disagree	3 (0.6)	0	

Potential Reasons Underlying Gaps in Ever Being Offered PrEP by 2019 (N = 536)			
		Never Offered PrEP (n = 242)	
Ever been to a Sisters clinic			
No	114 (21.3)	73 (64.0)	<0.001
Yes	422 (78.7)	169 (40.1)	
Tested for HIV in the past 6 mo			
No	124 (23.1)	70 (56.5)	0.001
Yes	412 (76.9)	172 (41.8)	

Potential Reasons Underlying Gaps in Ever Used PrEP by 2019					
	All Women	Never Used PrEP (n = 357; Row % of all Women)	*P* *	Not Using PrEP Currently (n = 474; Row % of all Women)	*P* *
Any condomless sex with regular partners in the past mo					
No	275 (51.3)	184 (66.9)	0.59	240 (87.3)	0.48
Yes	261 (48.7)	171 (65.5)		234 (89.7)	
Any condomless sex with clients in the past mo					
No	475 (88.8)	317 (66.7)	0.38	424 (89.3)	0.09
Yes	60 (11.2)	38 (63.3)		50 (83.3)	
Perceived risk of HIV in the next 12 months (N = 484)					
None/small	397 (82.0)	265 (66.8)	0.77	345 (86.9)	0.28
Medium/high risk	87 (18.0)	57 (65.5)		79 (90.8)	
I would receive support to take PrEP from: friends/peers					
Strongly agree	114 (21.4)	65 (18.3)	0.38	94 (82.5)	0.32
Agree	297 (55.6)	203 (57.2)		264 (88.9)	
Disagree	87 (16.3)	61 (17.2)		80 (92.0)	
Strongly disagree	36 (6.8)	26 (7.3)		34 (94.4)	
I would receive support to take PrEP from: sexual partners					
Strongly agree	70 (13.1)	39 (11.0)	0.22	56 (80.0)	0.24
Agree	228 (42.7)	149 (42.0)		201 (88.2)	
Disagree	157 (29.4)	112 (31.6)		143 (91.1)	
Strongly disagree	79 (14.8)	55 (15.5)		72 (91.1)	
I would receive support to take PrEP from: family					
Strongly agree	100 (18.7)	57 (16.1)	0.24	82 (82.0)	0.10
Agree	249 (46.6)	166 (46.8)		217 (87.2)	
Disagree	109 (20.4)	74 (20.9)		100 (91.7)	
Strongly disagree	76 (14.3)	58 (16.3)		73 (96.1)	

* *P* value from the Wald test, adjusted for age and the two study sites.

**TABLE 3.** Understanding Reasons Underlying Gaps in the PrEP Cascade Through Qualitative Interviews (N = 43)

Cascade Step	
Women's knowledge of PrEP and where they can access these services	They [Sister's clinic] advised me to use protection [condoms] and they told me about PrEP and its use...They told me to use it to prevent HIV infection and that I can get it at the New Start Center … I know of Umzingwane AIDS Council, CeSHHAR, Bekezela, and AVAC that deal with PrEP. (22-year-old, site A) **BYOLN05R1**
	They told me about it [PrEP]. I heard that they are pills that protect you from getting HIV even if you have unprotected sex with an infected person. I even heard about it on the radio.... They offered it [PrEP] to us but I said I would come when ready. (21-year-old, site A) **BYOCS24R1**
	We were educated about PrEP in groups and I immediately wanted it. We were given referral letters to start PrEP when we tested negative...It was near Blad Mission. (19-year-old, site B) **MTRLN04R1**
	I went to a place called [she forgot the name] … to have the pill [PrEP] that will prevent HIV infection. I cannot get infected by HIV if I have sex with someone with HIV while using PrEP every day (23-year-old, site B) **MTRCS41R3**
	Yes, I accompanied a friend to FACT and the other to PSI they wanted pills, the PrEP pills. (19-year-old, site B) **MTRCS37R1**
	I then went with him to New Start Center since he wanted to be tested with his girlfriend, so I asked them to explain about PrEP. (23-year-old, site B) **MTRCS22R1**
	The only place where they offered me PrEP was at Sisters where they encouraged me to take it but I did not. (22-year-old, site A) **BYOLN03R2**
Barriers to being offered PrEP	They offered it to me, but something came up and I could not manage to go, I got really busy (18-years-old, site B) **MTRLN08R3**
	I want to take PrEP, but time would not allow me, I am too busy to go to the clinic for this. (22-year-old, site A) **BYOLN03R1**
Reasons why women never started PrEP	It is not because I did not want, but I realized that there was no need for me to use it [PrEP] because I do not have unprotected sex. My boyfriend does not live in Zimbabwe, so I do not have unprotected sex. (18-year-old, site A) **BYOCS42R3**
	I did not like them …I do not like taking tablets. (20-year-old, site B) **MTRLN14R1**
	She explained and asked me whether I wanted to be referred for PrEP and then I said no because I was afraid that my mother would see the pills and she will ask me about those pills. If she asks that then it will be hard for me to stay at home because she will know about business [sex work] (19-year-old, site B) **MTRLN09R1**
	I told them not to refer me. I will continue to use condoms. (19-year-old, site B) **MTRLN09R1**
	The only place where they offered me PrEP was at Sisters [Sex Worker Clinic] where they encouraged me to take it [PrEP]. But I did not [take PrEP] because the clinic is a bit far from where I live and work. The problem was the time to commit to travel there because when they wanted me to go to PSI, I was also needed at Kelvin where I work. (22-year-old, site A) **BYOLN03R2**
	What if my mother sees it [PrEP] I do not want her to see such things that is why I refused the pills.(19-year-old, site B) **MTRLN09R2**
	I do not like taking pills. So when I heard of pills I lost interest….to be honest I forgot if they prevent [HIV] or not, because I did not have any interest in them. (20-year-old, site B) **MTRLN14R2**
	I do not really have problems taking them [pills], it is just that I initially did not understand the way they are used. When I eventually did, I realized just do not want to take them because I only use condoms to protect myself. (20-year-old, site A) **BYOLN04R3**
	I do not think I will be able to take it [PrEP] every day (19-year-old, site B) **MTRCS40R3**
	To be fair, I will not get sick if I do not take PrEP so I do not take it. Another reason is that at home my mother does not know that this is what I do [sex work], so she might find them and she will ask me what these pills are for and we might argue about it. Also, I fear that I may forget to take them and end up contracting HIV. So, I decided not to take the pills as I would rather use a condom... So I have actually started liking a condom. (18-year-old, site B) **MTRCS18R1**
	I am negative. I wanted to take PrEP but the processes are too tedious and long for me, I fear that I may also forget to take the tablets [PrEP]. Another thing that got me worried is that when I told him [boyfriend] that PrEP protects me from HIV he asked me where the HIV would be coming from and he asked why I wanted to take it alone and exclude him. So I got scared. (20-year-old, site A) **BYOCS33R2**
	I use condoms a lot and they hardly break for me, so I have not really been actively considering PrEP. (19-year-old, site B) **MTRLN09R2**
	I remember being told about it [PrEP], but I did not listen much about it….I did not have any interest getting to know more about the service, to be honest….I thought it was one of those useless programs. (23-year-old, site B) **MTRCS22R1**
Reasons why women stopped taking PrEP	I used to skip taking them [PrEP] and then I eventually stopped because I was going to school and feared that they would affect me, I often had headaches after taking them and I was forced to sit down and rest making others suspicious at school. I am also not doing sex work anymore because I have gotten back in school and I am now busy with my school. (18-year-old, site B) **MTRLN10R1**
	It is discouraging to repeatedly visit the clinic to get medication when you are not sick, so lost interest on PrEP. Another thing is that I never have sex with anyone without protection [condom], I felt like there is nothing it [PrEP] will help me with because I am using protection (21-year-old, site B) **MTRLN12R1**
	I did not like taking them [PrEP] ... They were too many and I decided to stop taking them… They were giving me a tummy ache. (20-year-old, site B) **MTRCS11R2**
	I thought it is better to use condoms instead of the pills because I feared that I could not adhere to them. (18-year-old, site A) **BYOCS26R1**
	I do not think I have had unprotected sex with any infected person, I use protection all the time. I felt that it [PrEP] has no use for me (19-year-old, site B) **MTRCS32R2**
	I stopped taking my medication [PrEP] last mo because I used to feel dizzy after taking them. I would have headaches to such an extent that I could not see, then I would be forced to sit down. It used to happen only for about five minutes but it was terrible. I also experienced a rash it was also bad. (23-year-old, site B) **MTRCS19R1**
	No particular reason. “*Hapana*” [nothing/no reason]. “*Hapana*” [I was neither motivated nor persuaded] (20-year-old, site B) **MTRLN16R2**
	No I have stopped now, I never went back there…Because I could not get transport money to get back and also I did not have enough time because I was writing exams and my aunt also did not have money for me to go to town. (19-year-old, site B) **MTRLN16R1**
	They said I should keep coming to get refills, so I just did not pursue it. It was too burdensome. (19-year-old, site B) **MTRCS32R2**
	I started in June and stopped. I went there [clinic] and they said that I should come back in July and I did not go back. I was in the rural areas; my father was ill. I then went back, and I was given the pills and I am now taking them [PrEP] (22-year-old, site A) **BYOCS43R3**

### Cascade Step 2: Ever Offered PrEP

In 2017, few women had been offered PrEP (11.7%, n = 112); by 2019, 54.9% (n = 294) reported ever being offered PrEP. *Never* being actively offered PrEP was lower among women who had been to a Sisters clinic (40.1% vs never: 64.0%) and had tested for HIV in the past six months (41.8% vs not tested 56.5%) (Table 2). The cost and opportunity cost of accessing PrEP, including missing school, limited women's ability to go to clinics where PrEP was available:

I was referred for PrEP in town but time does not permit...I do [want to take PrEP] but time won't allow me…Please bring some for me if you can. Because I really want it. (22 years) BYOCS25R1

Some women had been to the clinic but then were asked to come back the following day:

The first time I wanted to go and get PrEP I did not have the time to go there because of school. When I had the time and wanted PrEP, I was told to come the following day and never went back again. (23 years) MTRCS30R2.

### Cascade Step 3: Ever Taken PrEP

In 2017, 6.9% (n = 66) of women reported ever taking PrEP, increasing to 33.6% (n = 181) by 2019. There was little difference in PrEP use by perceived support for PrEP use and condomless sex in the past month (Table 2). PrEP use was strongly associated with ever being offered PrEP (60.5% vs 1.2%). In risk factor analyses (Table 4), ever using PrEP by 2019 was higher among women who, at enrollment, reported 10+ clients in the past month (44.5% vs 26.5% for 1–3 clients; adjOR = 1.71 95% CI: 1.06 to 2.76) and had been to a Sisters clinic in the past 12 months (54.5% vs 26.7%, adjOR = 2.92 95% CI: 1.91 to 4.46), with weaker evidence that use was higher among women who were ever/currently married (42.9% vs 29.1%, adjOR = 1.51 95% CI: 0.98 to 2.32).

**TABLE 4.** Enrollment Characteristics and Behaviors of Young Women Testing HIV-Negative and Followed up in 2019 and the Association Between These Factors and Self-Reporting Ever Using PrEP by 2019 (N = 538)

	Distribution of Characteristics and Behaviors (N, Column %)		Women Reporting Ever Using PrEP by 2019 (n, Row %)		Age-Adjusted and Site Adjusted OR (95% CI)	Adjusted OR (95% CI)	*P* ¶
Overall	538	100	181	33.6%	—		
Sociodemographics and history of selling sex at enrollment							
Age at time of survey (yr)							
18	109	20.3	34	31.2%	1.03 (0.94 to 1.13)	1.03 (0.94 to 1.13)	0.53
19	99	18.4	34	34.3%			
20	56	10.4	17	30.4%			
21	75	13.9	20	26.7%			
22	80	14.9	27	33.8%			
23	91	16.9	38	41.8%			
24	28	5.2	11	39.3%			
Marital status							
Single/never married	361	67.1	105	29.1%	1.00	1.00	0.06
Married/cohabiting/previously married	177	32.9	76	42.9%	1.51 (0.98 to 2.32)	1.51 (0.98 to 2.32)	
Educational attainment*							
No education/incomplete primary	15	2.8	8	53.3%	2.03 (0.71 to 5.86)	1.87 (0.65 to 5.42)	0.68
Complete primary education	32	6.0	12	37.5%	1.32 (0.61 to 2.85)	1.22 (0.56 to 266)	
Form 1–3	216	40.2	76	35.2%	1.12 (0.76 to 1.64)	1.06 (0.72 to 1.57)	
Form 4–6/college, cert, or degree	275	51.1	85	30.9%	1.00	1.00	
At risk of common mental health disorders†							
No	331	61.5	102	30.8%	1.00	1.00	0.24
Yes	207	38.5	79	38.2%	1.34 (0.92 to 1.94)	1.26 (0.86 to 1.83)	
Whether YWSS considered herself an FSW† (N = 532)							
No	167	31.4	53	31.7%	1.00	1.00	0.82
Yes	365	68.6	126	34.5%	1.05 (0.70 to 1.58)	1.05 (0.70 to 1.58)	
Age started selling sex†							
10–14	26	4.8	10	38.5%	1.13 (0.48 to 2.66)	1.10 (0.46 to 2.59)	0.80
15–17	250	46.6	80	32.0%	1.00	1.00	
18–19	150	27.9	53	35.3%	1.00 (0.63 to 1.58)	0.97 (0.61 to 1.54)	
20–24	111	20.7	37	33.3%	0.81 (0.46 to 1.44)	0.77 (0.43 to 1.38)	
Duration of selling sex†							
<2	136	25.3	33	24.3%	0.53 (0.33 to 0.85)	0.51 (0.32 to 0.83)	0.02
2–3	236	44.0	89	37.7%	1.00	1.00	
4+	165	30.7	58	35.2%	0.83 (0.53 to 1.31)	0.86 (0.54 to 1.35)	
No. of clients in the past mo‡							
1–3	219	40.7	58	26.5%	1.00	1.00	0.09
4–9	173	32.2	58	33.5%	1.39 (0.90 to 2.17)	1.24 (0.78 to 1.96)	
10+	146	27.1	65	44.5%	2.17 (1.37 to 3.42)	1.71 (1.06 to 2.76)	
Knowledge of HIV, perceived HIV risk support for PrEP use, and access to Sisters services at enrollment							
Knowledge of HIV prevention†							
No	278	51.7	97	34.9%	1.00	1.00	0.72
Yes	260	48.3	87	32.3%	0.90 (0.63 to 1.30)	0.94 (0.65 to 1.35)	
Perceived risk of HIV in the next 12 months (excluding women self-reporting a positive HIV test result at the last test, 37 missing data)§							
No chance: no possibility of becoming infected	94	19.8	35	37.2%	0.86 (0.50 to 1.47)	0.96 (0.54 to 1.71)	0.79
Small chance: could happen but not likely	178	37.5	64	36.0%	1.00	1.00	
Moderate chance: some possibility of becoming infected	71	15.0	22	31.0%	0.80 (0.44 to 1.45)	0.83 (0.44 to 1.55)	
High chance: likely to become infected	96	20.2	34	35.4%	1.02 (0.60 to 1.72)	1.06 (0.60 to 1.87)	
I do not know	36	7.6	11	30.6%	0.66 (0.30 to 1.44)	0.63 (0.27 to 1.44)	
I would receive support for PrEP use from: friends/peers (N = 500)‖							
Agree/strongly agree	401	80.2	148	36.9%	1.00	1.00	0.03
Disagree/strongly disagree	99	19.8	24	24.2%	0.63 (0.38 to 1.05)	0.54 (0.31 to 0.95)	
I would receive support for PrEP use from: sexual partner (N = 487)‖							
Agree/strongly agree	271	55.7	88	32.5%	1.00	1.00	0.40
Disagree/strongly disagree	216	44.3	72	33.3%	1.28 (0.86 to 1.91)	1.21 (0.77 to 1.92)	
I would receive support for PrEP use from: family (N = 498)‖							
Agree/strongly agree	331	66.5	124	37.5%	1.00	1.00	0.30
Disagree/strongly disagree	167	33.5	45	27.0%	0.67 (0.44 to 1.02)	0.79 (0.50 to 1.23)	
Had attended a Sisters clinic in the past 12 months†							
No	404	75.1	108	26.7%	1.00	1.00	<0.001
Yes	134	24.9	73	54.5%	3.37 (2.24 to 5.09)	2.92 (1.91 to 4.46)	
Self-identification as a sex worker and offer of PrEP between 2017–2019							
How YWSS identified throughout the study§							
Never identified as an FSW	105	19.8	28	26.7%	0.62 (0.37 to 1.05)	0.89 (0.51 to 1.57)	0.11
Did not identify as an FSW at enrollment, did at follow-up	61	11.5	25	41.0%	1.13 (0.63 to 2.02)	1.46 (0.78 to 2.73)	
Identified as an FSW at enrollment, not in follow-up	109	20.6	27	24.8%	0.54 (0.33 to 0.90)	0.61 (0.36 to 1.04)	
Identified as an FSW throughout the study	255	48.1	99	38.8%	1.00	1.00	
Ever offered PrEP between 2017–2019							
No	242	45.2	3	1.2%	—	—	<0.001
Yes	294	54.8	178	60.5%			

FSWs–female sex workers; OR–odds ratio; All models adjusted for age and site.

* Additionally adjusted for marital status.

† Additionally adjusted for marital status and number of year selling sex.

‡ Additionally adjusted for marital status, number of year selling sex, and attended a Sisters clinic in the past 12 months.

§ Additionally adjusted for marital status, number of year selling sex, attended a Sisters clinic in the past 12 months, and number of clients in the previous mo.

‖ Additionally adjusted for marital status, year selling sex, attended a Sisters clinic in the past 12 months, and perceived support for PrEP use from family/friends.

¶ From the Wald test.

PrEP use was lower among women who, at enrollment, reported selling sex for <2 years (24.3% vs 2–3-years: 37.7%, adjOR = 0.51 95% CI: 0.32 to 0.83) and who disagreed that friend/peers would support their PrEP use (24.2% vs 36.9%; adjOR = 0.54 95% CI: 0.31 to 0.95). Half the women self-identified as FSWs throughout the study (48.1%, n = 255). There was borderline evidence that PrEP use was associated with self-identification: use was lowest among women who never identified as an FSW (26.7%, n = 28/105) and who identified as an FSW at enrollment but not at follow-up (25.0%, n = 27/109). In qualitative interviews, women revealed a willingness to take PrEP but had concerns that it would disclose their sex work.

Ok, I did not want it because if I get it [PrEP] and take it home, my family would like to know why I have PrEP and what it is for, exposing my business [sex work] (22 years) BYOLN06R1.

Initiating PrEP was hindered by broader structural factors such as stigma, lack of support for PrEP use, alongside financial and opportunity costs of traveling to the clinic, and related to how PrEP was offered and needs to be taken:

…I wanted to take PrEP but the processes are too tedious and long for me, I fear that I may also forget to take the tablets [PrEP]. Another thing that got me worried is that when I told him [boyfriend] that PrEP protects me from HIV he asked me where the HIV would be coming from and he asked why I wanted to take it alone and exclude him. So I got scared. (20 years) BYOCS33R2

### Cascade Step 4: Currently Taking PrEP

In 2019, current PrEP use remained low at 11.5% (n = 62). Among women not currently using PrEP, a higher percentage (strongly) disagreed that they would receive support for PrEP use from family (Table 2). Reasons for discontinuing PrEP use were varied (Table 3) and included side effects and, as with PrEP use, reflected broader structural factors:

When my brother found the pills [PrEP] he got very cross, he yelled and beat me up. He asked me why I had not disclosed my [HIV] status to him. This was because he was not educated on PrEP [assumed she was HIV-positive]. My grandmother, however, knew that I was taking PrEP … She was fine with it. The way my brother was shouting attracted everyone's attention and the neighbors heard all his insults about him saying I am sick. That made me suicidal and I also decided to stop taking it. (24 years) BYOLN02R2.

Discontinuation of PrEP was also driven by perceived low HIV risk behaviors and recognizing the need to adhere to taking PrEP daily, leading some women to prefer condoms:

I thought it is better to use condoms instead of the pills because I feared that I could not adhere to them. (18 years) BYOCS26R1.

One woman described many reasons for discontinuing PrEP and later tested HIV-positive:

When I tested HIV-negative, they told me about the PrEP … So I got my PrEP for three months and then I stopped. I stopped taking the pills for many reasons. First, it was cumbersome and burdensome to take the pills every day and when I was not ill. Secondly, I felt more hungry than before, and lastly my brother was suspecting that I was on ART for HIV … so I was increasingly embarrassed about taking them [PrEP] ... after stopping I tested HIV-positive … (19 years) MTRCS20R1.

## DISCUSSION

After introduction of PrEP in the two DREAMS sites, just over half the women in our study reported ever being offered PrEP by 2019 and one-third ever used PrEP. Use was higher among women reporting an active offer of PrEP and was associated with factors likely to influence HIV risk, including being married/cohabiting, number of years selling sex, and reporting higher number of clients. Our mixed method analysis revealed that lack of support for use, costs and opportunity cost to travel to the clinic, side effects, and daily pill taking as reasons for observed gaps in the PrEP cascade.

Our study is subject to limitations. Data on an offer of PrEP use and current PrEP use were self-reported and therefore subject to error.^20^ Almost half the women recruited were lost to follow-up. Our outcomes are subject to bias if PrEP knowledge and use differ by follow-up. Our risk factor analysis may be subject to bias if PrEP use is differentially misreported by the variables explored. However, as our explanatory variables were measured at enrollment and most women had not yet used PrEP, we consider the risk of bias low. Despite limitations, we recruited and followed up more than 500 YWSS, who are underrepresented in research on FSWs. Furthermore, we used data from two time points, with our explanatory variables measured before most PrEP use.

Reported non-PrEP use was strongly associated with not having been offered it. In Kenya, Were et al^21^ found that, in the first two years of PrEP rollout (2017–2019), low provider screening for PrEP eligibility was among the most common missed opportunities for delivering PrEP to adolescent girls and young women. In South Africa, a study among FSWs and men who have sex with men found that 57% of individuals who had never used PrEP had never been proactively offered it.^18^ Fear of side effects was the primary reason for declining use.^18^ Similar to findings from our qualitative analysis, Emmanuel et al (2020)^22^ found that costs and frequency of HIV testing were barriers to PrEP uptake among FSWs in Nigeria. Using qualitative data collected during the SEARCH trial in Kenya and Uganda, Camlin^23^ et al (2020) found that daily pill taking was considered a bigger burden than HIV risk. Integrating PrEP into family planning, sexual and reproductive health, and targeted services is critical to increase opportunities to offer PrEP.^24^ With growing evidence of injectable PrEP efficacy,^25^ longer-acting PrEP may remove some supply-side barriers and those related to confusion between PrEP and ART.

In our study, a higher percentage of women offered PrEP initiated it than in the SEARCH trial, where 27% of high-risk individuals initiated PrEP.^17^ Our risk factor analysis found no evidence that self-perceived HIV risk at enrollment was associated with PrEP use. However, PrEP use was highest among women who identified as a FSW throughout the study and at follow-up, higher among women reporting more clients and selling sex for longer. Similarly, our qualitative findings underscore that women's decision making around PrEP includes consideration of risk based on sexual behaviors, including condom use and their perception of different sexual partners, with higher use among married/cohabiting women. These findings are similar to those from a qualitative study among serodiscordant couples in Zimbabwe, which found that risk perception was linked to willingness to take PrEP.^26^ These findings suggest that women in longer-term, stable relationships—who may find condom negotiation harder or condom use less pleasurable—–are seeking alternative prevention measures. Rather than focus on reduced HIV risk and medicalize PrEP, efforts to increase demand should focus on messaging around personal protection, reduced sex-related anxiety, and increased sexual pleasure with regular partners.^27,28^

Seroconversion after 24 months was similar by whether women reported ever using PrEP in and by 2019.^13^ Although we did not collect adherence data and our measures of ever and current PrEP use were self-reported, some women likely seroconverted because of inadequate adherence and/or PrEP discontinuation. A systematic review of 41 studies, representing 22,034 individuals, found high PrEP discontinuation at 1 month.^29^ Among the women in our study, low continued use of PrEP was likely driven by a combination of supply-side reasons and structural factors, including stigma related to HIV and sex work, association of medication with ART, and lack of social support.

Similar to our findings, qualitative data in the SEARCH trial identified that, in addition to pill burden, PrEP use was discontinued because of HIV-related/ART-related stigma, relationship dissolution, and lack of support for use.^23^ A Zimbabwean study among FSWs living with HIV found that women experienced more stigma related to their sex work than HIV stigma;^30^ a systematic review of 15 studies from sub-Saharan Africa identified stigma related to sex work and HIV as a barrier to HIV testing.^31^ Addressing stigma associated with sex work, as well as HIV, may support women's PrEP use and adherence. Family support emerged during qualitative interviews and quantitative findings suggest lower PrEP use among women with less support from friends/peers, with levels of PrEP use by perceived family support similar to those reported by perceived support from friends/peers. These findings align with evidence from a scoping review of ART uptake and adherence among FSWs, which found that social support facilitated uptake and adherence.^32^ Again, as PrEP becomes available in alternate and longer-term formulations, these barriers may subside. However, strategies to address HIV-related/ART-related stigma and increase support for PrEP use remain critical.

## CONCLUSION

To date, there are little data on PrEP use over time among YWSS, including young FSWs aged <25 years. As PrEP is scaled-up across southern and eastern Africa, understanding barriers to use among this group of women is critical to support uptake and adherence.^3,23^ Our study shows that barriers to PrEP initiation and continuation are similar to those among other populations and in other countries. To increase effectiveness among YWSS, proactive offers of PrEP are needed combined with building social support among peers, partners, and communities as knowledge and understanding of PrEP increases. Messaging to encourage PrEP use should position PrEP in a way that matches women's perceptions regarding what places them at risk of HIV.

## Supplementary Material

SUPPLEMENTARY MATERIAL
