# Supplementary material for: How Can We Support the Use of Oral PrEP Among Young Women who Sell Sex? A PrEP Cascade Analysis
Source: J Acquir Immune Defic Syndr. 2021 May 19;88(1):45–56. doi: 10.1097/QAI.0000000000002733 (PMC8357043; doi:10.1097/QAI.0000000000002733)
Supplement: SUPPLEMENTARY MATERIAL [file qai-88-45-s001.docx]

**Supplemental Table 1. HIV sero-conversion between 2017 and 2019 by steps in the PrEP cascade (N=538)**

|  | **Distribution of outcome**  **(N, col%)** | **Number of women sero-converting by 2019**  **(n, row%)** | **p-value*** |
| --- | --- | --- | --- |
| **Ever heard of PrEP by 2019** |  |  |  |
| No | 23 (4.3) | 2 (8.7) | 0.58 |
| Yes | 514 (95.7) | 28 (5.5) |  |
| **Ever offered PrEP by 2019** |  |  |  |
| No | 242 (45.2) | 10 (4.1) | 0.26 |
| Yes | 294 (54.9) | 19 (6.5) |  |
| **Ever taken PrEP by 2019** |  |  |  |
| No | 357 (66.4) | 19 (5.3) | 0.47 |
| Yes | 181 (33.6) | 12 (6.6) |  |
| *p-value is from Wald test and adjusting for age and site; | | | |
